# The Neurobiology of Infant Attachment-Trauma and Disruption of Parent–Infant Interactions

**DOI:** 10.3389/fnbeh.2022.882464

**Published:** 2022-07-22

**Authors:** Nimra Naeem, Roseanna M. Zanca, Sylvie Weinstein, Alejandra Urquieta, Anna Sosa, Boyi Yu, Regina M. Sullivan

**Affiliations:** ^1^Department of Psychology, Center for Neuroscience, New York University, New York, NY, United States; ^2^Emotional Brain Institute, The Nathan S. Kline Institute for Psychiatric Research, Orangeburg, NY, United States; ^3^Child and Adolescent Psychiatry, New York University Langone School of Medicine, New York, NY, United States

**Keywords:** adversity, trauma bonding, attachment, social buffering, child abuse, mother-infant dyad, stress, hypothalamic-pituitary-adrenal axis

## Abstract

Current clinical literature and supporting animal literature have shown that repeated and profound early-life adversity, especially when experienced within the caregiver–infant dyad, disrupts the trajectory of brain development to induce later-life expression of maladaptive behavior and pathology. What is less well understood is the immediate impact of repeated adversity during early life with the caregiver, especially since attachment to the caregiver occurs regardless of the quality of care the infant received including experiences of trauma. The focus of the present manuscript is to review the current literature on infant trauma within attachment, with an emphasis on animal research to define mechanisms and translate developmental child research. Across species, the effects of repeated trauma with the attachment figure, are subtle in early life, but the presence of acute stress can uncover some pathology, as was highlighted by Bowlby and Ainsworth in the 1950s. Through rodent neurobehavioral literature we discuss the important role of repeated elevations in stress hormone corticosterone (CORT) in infancy, especially if paired with the mother (not when pups are alone) as targeting the amygdala and causal in infant pathology. We also show that following induced alterations, at baseline infants appear stable, although acute stress hormone elevation uncovers pathology in brain circuits important in emotion, social behavior, and fear. We suggest that a comprehensive understanding of the role of stress hormones during infant typical development and elevated CORT disruption of this typical development will provide insight into age-specific identification of trauma effects, as well as a better understanding of early markers of later-life pathology.

## Introduction

We have known for decades that early-life adversity during a sensitive period robustly increases vulnerability to physical and mental disorders, as has been replicated in other animal species (Sorokin, 1942; Scott, 1949; Wolff, 1949; Dollard and Miller, 1950; Selye, 1950; Lehmann, 1952; Bowlby, 1953, 1958; Beach and Jaynes, 1954; Fisher, 1955; Levine et al., 1957; Lorenz, 1958; Hess, 1959; Denenberg and Whimby, 1963; Hinde and Spencer-Booth, 1971; Cicchetti and Toth, 1995; Evans and Porter, 2009; Chen et al., 2021; Tronick et al., 2021). Since then, a robust literature has emerged documenting the ubiquitous impact of early-life adversity on the later-life brain, encompassing almost every level of analysis ranging from genes and epigenetics, to neurotransmitters and hormones, to systems-level neuroscience with region of interest (ROI) analysis across diverse altricial species (Kim et al., 1999; Kaufman et al., 2000; McEwen, 2003; Fish et al., 2004; de Kloet et al., 2005; Rogosch and Cicchetti, 2005; Andersen et al., 2008; DiCorcia and Tronick, 2011; Bliss-Moreau et al., 2013; Khan et al., 2015; Tost et al., 2015; Tronick and Hunter, 2016; Doherty et al., 2017; Demers et al., 2018; Lester et al., 2018; Reck et al., 2018; Teicher, 2018; Gur et al., 2019; Perry et al., 2019; Sullivan and Opendak, 2020; Smith and Pollak, 2021). It is well established that while some adversity can produce resilience and adaptive behaviors to later-life harsh conditions, repeated exposure to early-life adversity goes beyond this adaptive programming to produce maladaptive behaviors and psychiatric disorders (Stevens et al., 2009; Daskalakis et al., 2013; Meyer and Lee, 2019; Yu et al., 2019; Ellis et al., 2020; Frankenhuis et al., 2020; Nelson et al., 2020; Sideli et al., 2020; Boyce et al., 2021). While it has become clear that repeated adversity immediately impacts the child’s brain during infancy (Graham et al., 1999, 2015, 2016; Tottenham, 2013), what has remained elusive is our understanding of the acute impact of repeated infant adversity during infancy, especially in social vs. non-social contexts and its causal mechanism.

## Infant Trauma Within a Social Context (Attachment) and Emergence of Later-Life Pathology

**FIGURE 1:**
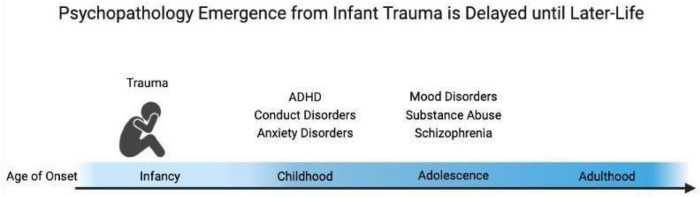
Early-life trauma experiences produce delayed expression of pathology across the lifespan. The age of onset of symptoms of anxiety, ADHD, and conduct disorders appears in childhood, while substance abuse, schizophrenia, and mood disorders usually begin to slowly emerge around adolescence.

As first noted by Bowlby, attachment forms regardless of the quality of caregiving. However, trauma associated with the attachment figure has been emphasized as particularly influential in vulnerable contexts associated with later-life pathology (Canetti et al., 1997; Maestripieri, 1998; McEwen, 2003; Zeanah et al., 2003; Ziabreva et al., 2003; Adam et al., 2004; Benoit, 2004; Fish et al., 2004; Pryce et al., 2004; Lyons-Ruth, 2008; Gunnar et al., 2015; Callaghan and Tottenham, 2016; Gee, 2016; Callaghan B. L. et al., 2019; Erickson et al., 2019; Opendak et al., 2019; Perry et al., 2019; Raineki et al., 2019; Tottenham et al., 2019; Twohig et al., 2021). Indeed, self-reports of patients who had experienced early-life adversity in the context of “interpersonal violation” had the highest level of self-reported depression and anxiety symptoms, suggesting the importance of social context in the experience of a traumatic event (Chu et al., 2013). Additional research suggests that the impacts of trauma on the developmental trajectory slowly emerge throughout the lifespan: while anxiety emerges early in development, most other psychiatric disorders emerge around peri-adolescence as illustrated in Figure 1 (Gurley et al., 1996; Weems and Costa, 2005; Ghandour et al., 2019).

The delayed onset of psychopathology induced by early-life trauma has presented the challenge of identifying the early-life roots of later-life psychiatric disorders. However, more recently, an emphasis on the subtle age-specific expression of later-life psychiatric disorders suggests there are earlier symptoms of dysfunction that serve as markers of later-life pathology (Shear et al., 2006; Bittner et al., 2007; Pine, 2007; Zeanah et al., 2016; Bushnell et al., 2020; Creswell et al., 2020; Gold et al., 2020; Ciuhan and Iliescu, 2021; Cuijpers et al., 2021; Donmez and Ucur, 2021; Havens et al., 2021; Smith and Pollak, 2021). For example, one reliable early-life marker of later-life pathology is observable through the increased expression of fear and anxiety, which is sometimes diagnosed as an anxiety disorder (Jovanovic et al., 2014; Grossmann and Jessen, 2017; Creswell et al., 2020). Notably, there is typically a social context to this early-life fear and anxiety, observable through excessive fear of strangers, novel environments, and distress upon separation from caregivers (i.e., Separation Anxiety) and this is damaging enough to disrupt typical, daily childhood experiences (Lebowitz et al., 2018). These early-life altered behaviors are correlated with ubiquitous alterations in the brain, including limbic brain circuits important in emotion, cognition, and stress regulation (Cicchetti and Toth, 1995; Graham et al., 1999; Teicher et al., 2002; Grossman et al., 2003; Carpenter et al., 2004; Green et al., 2010; Sher and Vilens, 2010; Schumann et al., 2011; Di Martino et al., 2014; Rinne-Albers et al., 2015; Hanson et al., 2018).

To further tease apart the correlations between early-life adversity and a social context with later-life pathology, we look to animal research with a translational approach. While some may suggest that parent–infant attachment is unique to humans, John Bowlby, the father of Attachment Theory, was strongly influenced by animal research on attachment. Bowlby’s discussions with animal researchers, specifically with ethologists studying rodents, non-human primates, and avian imprinting (Lorenz, 1958; Harlow and Zimmermann, 1959; Hess, 1959; Kovach and Hess, 1963; Seay and Harlow, 1965; Hinde and Spencer-Booth, 1971; Suomi et al., 1971; Lorenz et al., 1982; Hinde, 2005; van der Horst et al., 2008), led him to postulate that attachment is phylogenetically preserved across species, further supporting the usefulness of cross-species research to inform the understanding of child development. Despite the fact that animal research cannot capture the complexity of the child’s developmental experiences, it can permit us to identify homologous brain networks and circuits observed in both animals and humans that support the fundamental features of the attachment circuit by performing invasive procedures that probe into causal mechanisms of typical and atypical attachment.

In Attachment Theory, Bowlby highlights that the quality of care an infant receives establishes the quality of attachment, and thus consequently the infants’ social interaction with the parent (Spitz, 1960; Bowlby, 1965, 1969; van der Horst et al., 2008). While it has been historically thought that the infant’s attachment to the caregiver was innate, we now understand attachment is a learned process that can occur between non-biologically related individuals, examples of which can be seen in cases of adoption of young infants (van den Dries et al., 2009; Raby and Dozier, 2019). Using ethologists’ research on avian imprinting in chicks, Bowlby postulated that children also had an attachment learning circuit within their brain. Specifically, research in newly hatched chicks showed learning was an evolutionary mechanism supporting bonding with the parent. This provided a framework that permitted early attachment researchers to consider new features of attachment in children. For example, imprinting research showed that the critical feature of attachment learning was not based on a specific parental care behavior, but rather through simple movement of the infant’s attachment target (Hess, 1959). Furthermore, even though there is a biological predisposition for attachment to bird-like objects, the moving object did not need to approximate a caregiving behavior, and the moving object could be almost any object (i.e., box, another species) to support attachment learning in the chick (Sambraus and Sambraus, 1975; Beaver et al., 1976). Moreover, most surprisingly, during this sensitive period of imprinting, shocking chicks actually produced an attachment to the moving attachment figure (Hess, 1964; Sullivan, 2012), further illustrating that attachment forms regardless of the quality of caregiving, including in the experience of pain.

While this mechanism of attachment in imprinting may seem counterintuitive, from an evolutionary viewpoint, selection pressure likely favored robust features of the attachment figure and increased the probability of a chick’s survival. Overall, this research supports the notion that regardless of the quality of parental care received, an infant will form an attachment. As will be discussed in more detail below, this association of pain and attachment in infants can also be seen in rodents, dogs, non-human primates, and children, where abusive parental care supports robust attachment (Kim et al., 1999; Rincón Cortés et al., 2013). In humans, diverse caregiving across different cultures all support attachment between the child and caregivers, suggesting that the evolutionary pressures on human attachment likely did not focus on one specific parental behavior to support attachment formation (Bornstein et al., 2017; Abraham and Feldman, 2018; Keller, 2018; Lansford, 2021). Within this view, it is not too surprising that low levels of parental care (deprivation, neglect) and maltreatment from the caregiver both support attachment (Cicchetti and Toth, 1995; Raineki et al., 2010; Granqvist et al., 2017). Furthermore, it is important to highlight that parental care is not one behavior, but many behaviors, each with specific functions, including nurturing, feeding, protecting, retrieval, and grooming, each with their own neural circuitry in both males and females (Numan, 1988; D’Cunha et al., 2011; Wu et al., 2014; Elyada and Mizrahi, 2015; Kohl et al., 2017; Pawluski et al., 2017; Abraham and Feldman, 2018; Fang et al., 2018; Keller et al., 2019; Bridges, 2020; Maguire et al., 2020; Carcea et al., 2021).

In summary, research on both human and non-human animals describes a sensitive period in early life when attachment learning occurs regardless of the quality of care the infant receives, including nurturing and maltreatment. This attachment system has short-term benefits in that infants will always attach (Perry and Sullivan, 2014). Yet early-life maltreatment induces later-life ubiquitous detrimental effects on the brain and behavior, with the most prominent effects of early-life trauma delayed until peri-adolescence (Kessler et al., 2007; Schmelzle-Lubiecki et al., 2007; Rincón Cortés and Sullivan, 2010, 2014; Rincón Cortés et al., 2013; Tzanoulinou and Sandi, 2015; Perry et al., 2019; Murthy and Gould, 2020). While trauma experienced during the process of brain development can perturb the developmental process, mounting evidence also suggests that the processing of trauma within the infant attachment circuits may integrate trauma into attachment and alter emotional brain circuits.

## Stress Hormone Corticosterone Can Uncover Trauma-Induced Pathology in Infancy

The most notable impacts of this adversity are within a social context where children display heightened separation anxiety, decreased ability to soothe, and altered fear, all of which are associated with acute increase in stress hormones. Indeed, increased levels of stress hormones have consistently been shown to negatively impact neurobehavioral development (Cicchetti and Toth, 1995; Teicher et al., 2002; de Kloet et al., 2005; Bosquet Enlow et al., 2014; Rahman et al., 2016; Bonacquisti et al., 2020; Khoury et al., 2021). However, more recent literature has highlighted the importance of the parent (or other important caregiver) in blunting the infant’s stress hormone increase (i.e., social buffering). Specifically, research supports that secure attachment to caregivers provides a safe haven which can shield a child from the negative implications of increased stress and prevent it from compromising functioning, but infant attachment associated with a maltreating caregiver is associated with reduced social buffering (Cicchetti and Toth, 1995; Gunnar and Fisher, 2006; Fries et al., 2008; Evans and Porter, 2009; Pechtel and Pizzagalli, 2011; Gunnar et al., 2015; Hanson et al., 2015; McLaughlin et al., 2015; Sanchez et al., 2015; Drury et al., 2016; Koss et al., 2016; Howell et al., 2017; Raineki et al., 2019; Opendak et al., 2020; Perry et al., 2020; Shakiba and Raby, 2021; Smith and Pollak, 2021). In other words, one reason for high stress in infancy is due to infants’ experiencing trauma, but it is also due to the infants’ inability to use the parent to socially buffer their stress response—both of which can produce high levels of stress hormones during infancy.

To further investigate the role of stress in modulating response to adversity, experiments on experiences of adversity within varying caregiving contexts were conducted. Early research within child development showed that increasing stress could uncover disrupted behavior toward the caregiver, and this was capitalized upon by Ainsworth in the development of the Strange Situation Procedure (SSP) (Ainsworth and Bell, 1970). SSP was designed by Ainsworth to assess children’s style of attachment to a caregiver: after repeated separations and reunions with their caregiver and a stranger, the final reunion with the caregiver measured how well the child could be soothed by the attachment figure to define secure vs. insecure attachment.

An additional category was later added to Ainsworth’s attachment styles and termed disorganized attachment, a form of attachment in which the child exhibits intense parent-directed social behaviors that are contradictory, combined with expressions of confusion and apprehension, and immobility/freezing (Main and Solomon, 1990; Granqvist et al., 2017). Disorganized attachment is also the only attachment style linked to later-life pathology, indicating the importance of understanding the role it plays in the roots of mental illness (Jacobvitz and Hazen, 1999; Lyons-Ruth and Jacobvitz, 1999; Allen, 2001; Cassidy and Mohr, 2001; Bakermans-Kranenburg and van Ijzendoorn, 2009).

**FIGURE 2:**
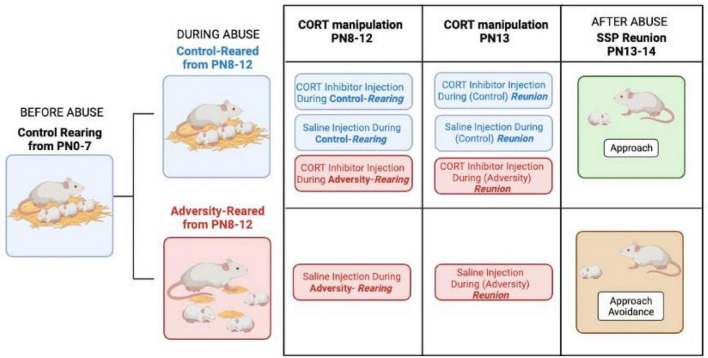
Using the Strange Situation Procedure (SSP) (Ainsworth, 1978), Mary Ainsworth was able to uncover behavioral pathology in children through a series of stressful separations and reunions of the child with the parent or a stranger. This was modeled in rat pups (Opendak et al., 2020) and illustrated the timeline of adversity-rearing with low bedding vs. control-rearing from postnatal (PN) 8–12 and testing using the rodent Strange Situation Procedure (SSP) at PN13-14 when adversity-reared pups’ stress hormone corticosterone (CORT) remains elevated. The adversity-rearing procedure provides the mother limited bedding and the inability to nest build, which induces pup rough handling (i.e., stepping on or dragging pups) but does not alter the level of nurturing care (Opendak et al., 2019). During the SSP test’s final reunion with the mother, control-reared pups stayed with the mother even when injected with a CORT inhibitor called metyrapone (MET) or saline. The MET also impacted social behavior in adversity-reared pups, by exhibiting approach behavior, almost reversing the impact of stress on the pup at the final SSP reunion. However, adversity-reared pups with naturally high levels of CORT, have reduced social interaction with the mother, which is highlighted by lack of approach behavior in the saline injection condition.

The rodent literature has shown that in the natural nest with the mother, adversity-reared pups still show typical social behavior with the mother, at least when not being actively maltreated (Opendak et al., 2020), suggesting similar to the human SSP, that potentially some stress is required to uncover pathology. To test this, we developed a rodent SSP test and directly compared adversity-reared rodent data with child SSP data collected on at-risk children in the lab of Mary Dozier (Opendak et al., 2020). As illustrated in Figure 2, from postnatal (PN) day 8 to 12, infant rats were adversity-reared by a rat mother provided with insufficient nest-building material, inducing rough handling of pups (i.e., stepping on and dragging pups, but nurturing behaviors stay at a similar level to control reared pups). At PN13-14, adversity-reared pups showed social deficits in the SSP’s last reunion with the mother, as evidenced by pups showing approach-avoidance behavior toward the mother. These rodent SSP behavioral results appear to coincide with the child’s SSP results, further providing a pathway for cross-species translational research to uncover causal mechanisms. To further understand the role of stress in uncovering infant markers of later-life pathology, cortical local field potentials (LFPs—telemetry system without cables, enabled undisturbed recordings) were measured and the variable of stress was investigated through pharmacological manipulation of corticosterone.

(CORT) during SSP’s last reunion with the mother (see Figure 2 phase after abuse). Specifically, the results from this experiment indicated that control-reared pups showed a dynamic LFP response to the mother (amygdala and cortical) (Sarro et al., 2013; Courtiol et al., 2018), but the adversity-reared pups exhibited blunted LFP response to both approaching and interacting with the mother (Opendak et al., 2020). To define causal mechanisms and explore the role of stress in uncovering pathology, we decreased adversity-reared pups’ stress hormones during the SSP, and this showed repair of the adversity-reared social behavior and LFP (Opendak et al., 2020). During the SSP, the rodent mothers were anesthetized, to ensure that maternal care could not account for the behavioral or neural differences between groups. With maturation and pups’ transition to independence, the maternal regulation of LFP in the infant’s brain begins to waneand highlights a major difference in the infant and adult brain’s response to the social environment. The age-specific ability of stress hormones and previous maternal care to modulate the patterns of LFP neural oscillations has profound implications, specifically because these early-life oscillations have been identified as a robust neural mechanism for brain programming (Katz and Shatz, 1996; Le Van Quyen et al., 2006).

It is important to note that prolonged adversity-rearing impacts many processes throughout the brain, and depending on the level of analysis indicated, it can either potentiate or attenuate the response to the mother (or caregiver). For example, imaging and optogenetic manipulation of pups following adversity-rearing suggest that pups’ amygdala is altered in a myriad of ways, including increases and decreases in the functionality of the basolateral amygdala (BLA) and the central amygdala (CeA), changes in neurogenesis, and an increase in neural activity as indicated by c-Fos, 2-DG autoradiograph and LTP/Ltd. (Thompson et al., 2008; Raineki et al., 2019; Opendak et al., 2021).

Furthermore, CORT during infancy has a role in switching the amygdala on and off. For example, young rats cannot be fear conditioned until they are about PN10 (Haroutunian and Campbell, 1979; Camp and Rudy, 1988; Sullivan et al., 2000), which is due to this conditioning procedure failing to recruit the amygdala in pups younger than PN10 (Sullivan et al., 2000). However, increasing CORT in pups younger than PN10 can permit amygdala-dependent fear learning, either through systemic or intra-amygdala microinjections (Moriceau and Sullivan, 2006; Moriceau et al., 2006) or by naturally increasing young pups CORT levels through adversity rearing (Plotsky et al., 2005; Walker et al., 2017). In addition, in pups older than PN10, when shock induces a CORT level high enough to permit amygdala plasticity, blocking CORT blocks fear learning: CORT can be blocked either systemically, with micro-infusion of a CORT blocker into the amygdala, by blockade of activation of the HPA axis at the level of the hypothalamus, and through maternal presence (Stanton et al., 1987; Richardson et al., 1989; Suchecki et al., 1993; Moriceau and Sullivan, 2006; Moriceau et al., 2006; Barr et al., 2009; Upton and Sullivan, 2010). This fear conditioning blockade by maternal presence has been replicated in children suggesting this CORT regulation of the amygdala and gating of emotional expression is potentially a global feature of fear in infants across altricial species (Callaghan B. L. et al., 2019).

In summary, CORT has a unique role in infant rats that gates emotional expression and learning, this is best observed by the slowly increasing levels during typical development and its permissive role in amygdala expression of fear around PN10. A precocious increase of CORT induced by adversity-rearing can derail this typical developmental trajectory through at least a few ways. First, atypically high levels of CORT in pups < PN10 permits the early expression of amygdala-dependent fear. Second, elevated CORT also engages the amygdala in young preweaning pups, a process that disrupts social behavior (Chareyron et al., 2012; Raper et al., 2014). Overall, developmentally, atypically high CORT levels can potentially disrupt the complex and delicate orchestration of pups’ age-dependent adaptation to life within the nest, including potential disruption of the pace of the path to independence.

## Parental Reduction of Threat-Induced Stress Increase (I.E., Social Buffering) Is Reduced in Infants With Adversity-Rearing

The ability of the parent to reduce their child’s response to a threat was first highlighted by Bowlby, who noted a child was more likely to explore a novel environment if the parent was present (Bowlby, 1958, 1965). Since then, work within developmental psychology has shown that this system involves the reduction of the threat induced increase in stress hormones (Gunnar and Donzella, 2002; Perry et al., 2016), a process sometimes termed social buffering (Kiyokawa and Hennessy, 2018). The ability of a parent to socially buffer (reduce stress hormones under threat) their offspring was first identified in infant rodents (Stanton et al., 1987; Suchecki et al., 1993) and quickly replicated in non-human primates (Hennessy et al., 2009), as well as in children where the level of social buffering is correlated with attachment quality and anxiety (Nachmias et al., 1996; Gunnar and Donzella, 2002; Gunnar and Quevedo, 2007; Gunnar et al., 2015; Tottenham et al., 2019).

**FIGURE 3:**
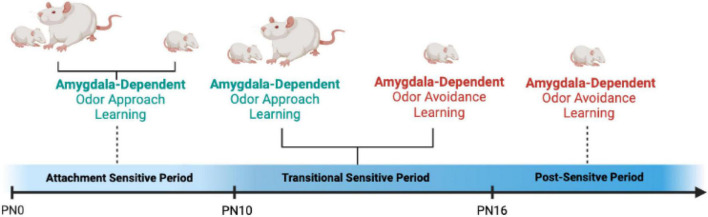
The development of amygdala-dependent odor-shock fear learning across infancy in rats is dependent upon infant’s endogenous shock-induced stress hormone CORT. From PN0 to PN9, when stress hormone levels are not increased by shock, pups do not engage the amygdala and cannot learn fear. Indeed, odor-shock conditioning engages the attachment circuit and pups learn to approach the odor previously paired with shock. This newly learned odor can replace the maternal odor and support nipple attachment and social interactions with the mother. At PN10, shock induces an increase in CORT and amygdala-dependent fear learning emerges. However, between the ages of PN10-PN15, maternal presence blocks pups shock-induced CORT release and pups do not learn amygdala-dependent fear. Instead, they revert back to the sensitive period learning and express an odor preference. This brief 5-day period is termed the “Transitional Sensitive Period,” because maternal presence temporarily turns off amygdala plasticity. At PN16, fear learning resembles “adult-like,” odor learning and amygdala plasticity is engaged regardless of maternal presence and CORT level (Sullivan et al., 2000; Moriceau and Sullivan, 2006; Moriceau et al., 2006; Barr et al., 2009).

Animal research has been helpful in defining causal mechanisms of social buffering, highlighting that maternal presence blocks the amygdala response to threat in infant rats, thus blocking plasticity in the brains’ core fear processing center as seen in Figure 3 (Sullivan et al., 2000; Moriceau and Sullivan, 2006; Moriceau et al., 2006; Barr et al., 2009). Capitalizing on the experimental ability to manipulate the amygdala to test causation in rodents, these studies show that between the ages of PN10-15, one could override maternal suppression of the amygdala with CORT microinfusions into the BLA, or controlling CORT at its source, the paraventricular nucleus (PVN) of the hypothalamus (Shionoya et al., 2007). This ability of the mother to alter pup’s amygdala-dependent fear wanes as the pup begins to prepare for independence.

Specifically, around PN16, while the mother continues to decrease pups’ threat-induced stress hormone increase, the mother can no longer suppress the amygdala’s plasticity (Upton and Sullivan, 2010). Furthermore, CORT’s action on the amygdala initiates an intracellular molecular cascade that directly impacts plasticity mechanisms that block the amygdala’s response to shock and prevents learning. As a result, the impact of maternal presence goes beyond the amygdala and impacts other brain networks important for fear learning, such as the ventral tegmental area (VTA) and prefrontal cortex (PFC) (Sullivan et al., 2000; Moriceau and Sullivan, 2006; Moriceau et al., 2006; Thompson et al., 2008; Barr et al., 2009; Upton and Sullivan, 2010; Opendak et al., 2018, 2019; Robinson-Drummer et al., 2019), which are required to support amygdala responsiveness and learning in early-life (Thompson et al., 2008; Ehrlich et al., 2012). Moreover, maternal presence blockade goes beyond the amygdala and fear learning: nursing blocks taste aversion learning before the age of functional emergence of the amygdala into this learning (Gubernick and Alberts, 1984; Shionoya et al., 2006) and impacts learning of the cerebellum-dependent eyeblink conditioning (Freeman and Nicholson, 2000; Claflin et al., 2021).

More recently, maternal suppression of fear learning has been replicated in children; 4-year-old children can learn fear, but fear learning is suppressed in the presence of the parent (Tottenham et al., 2019). Importantly, since maternal presence can block amygdala activation in the presence of a threat in children, there is a strong suggestion that the mechanism for suppressed learning was parental amygdala suppression (Tottenham et al., 2012; Gee et al., 2014). This complements research over the past decade, as numerous studies have documented the robust impact of maternal presence on the functioning of the child’s brain (Parma et al., 2013; van Rooij et al., 2017; Rogers et al., 2020).

In summary, within typical development across species, the presence of the caregiver reduces infants’ response to trauma by reducing the stress hormone release. Animal research suggests that the mother blocks the release of stress hormones by blocking activation of the HPA axis and depriving the amygdala of an age-specific dependence on the stress hormone CORT. CORT is required to engage plasticity mechanisms within the amygdala during the transitional sensitive period (see Figure 3), with the mother blocking its release, thus blocking the response to trauma, and learning about the trauma. The evolutionary significance of this maternal blockade of trauma-induced stress release and its blockade of responsiveness to trauma may be to protect the infant brain from the disruptive effect of stress hormones in early life (Sapolsky, 2009).

## Mechanisms of the Social Context of Trauma-Inducing Pathology

The child development literature suggests that the parent–infant social buffering system is degraded with repeated trauma-parent associations, which can occur in maltreatment (Nachmias et al., 1996; Gunnar and Donzella, 2002; Gunnar, 2003; Loman and Gunnar, 2010; Tottenham et al., 2010), and has been replicated in both rodent and non-human primates (Hostinar et al., 2014; Gunnar, 2017; Howell et al., 2017; Kiyokawa and Hennessy, 2018; Robinson-Drummer et al., 2019; Opendak et al., 2020; Chen et al., 2021; Claflin et al., 2021; Hilberdink et al., 2021; Keogh et al., 2021).

**FIGURE 4:**
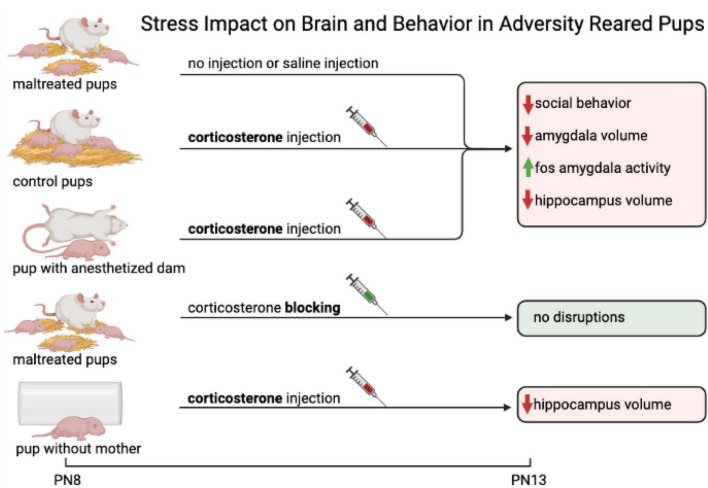
This figure illustrates early-life adversity. If early-life adversity occurs in the presence of the attachment figure, it disrupts neurobehavioral development equivalent to maltreatment (amygdala, hippocampus). While early-life adversity when the rat pup is alone has less impact (only hippocampus). The impact of stress on the brain and behavior in adversity-reared pups. With 5 different experimental conditions, one can tease apart the impact of parental care, parental presence, and impact of stress on pup development. Pups are assigned experimental conditions at postnatal day 8 (PN8). Pups in the first condition are adversity-reared (maltreated) and are given no injection or a saline injection. The second group of control pups is typically reared and receives a CORT injection. The third group of pups is in the presence of an anesthetized mother. The mother in this condition is not displaying any behavior but is still present and is neither delivering maltreatment nor nurturing care. These pups also receive a CORT injection. The fourth group is another maltreated pup condition and receives a CORT blocker. The fifth group of pups are alone (no maternal presence) and receive a CORT injection. Following varying manipulations, pups are assessed at PN13. Maltreated pups, Control pups, and pups with anesthetized mother all show similar impacts on behavior and brain functionality: social behavior is decreased, amygdala volume is decreased, c-Fos amygdala activity is increased, and hippocampus volume is decreased. The fourth group of maltreated pups who receive CORT blocking, show no disruption in behavior or brain functionality. The fifth group of pups without mother shows decreased hippocampal volume. This suggests that the amygdala is sensitive to repeated exposure to high levels of CORT and adverse experiences, whereas the hippocampus is not.

To better understand how the quality of caregiving can alter the infant’s neurobehavioral response to trauma, we again can look to research on rodents. As illustrated in Figure 4 early-life adversity, if it occurs in the presence of the attachment figure, disrupts neurobehavioral development equivalent to maltreatment (amygdala, hippocampus), while early-life adversity when the rat pup is alone has less impact (only hippocampus). Specifically, we investigated the maternal impact on the neurobehavioral responses of adversity-reared pups by using a shortened 90 min/day adversity-rearing paradigm for 5 days (PN8-12), as this was sufficient to produce the pathological effects of low bedding adversity rearing manipulations (Strüber et al., 2014; Doherty et al., 2017; Walker et al., 2017). This shortened procedure enabled repeated pharmacological increases or decreases of pup’s CORT levels, including increasing the CORT during control-rearing and blocking CORT during adversity-rearing—thus isolating CORT’s impact on neurobehavioral responses of infants independent of maternal care experience (Raineki et al., 2019; Opendak et al., 2020). This experiment further deconstructed the role of the mother by removing maternal behavior and permitting pups to interact with an anesthetized mother, or just an inanimate object, while also manipulating pups’ CORT levels (Raineki et al., 2019). As illustrated in Figure 4, pups were tested at PN13 for social behavior toward the mother, as well as amygdala and hippocampal deficits (volume, neurogenesis, c-Fos, LFP). All groups with increased CORT (either repeated injections of CORT or naturally elevated from adversity-rearing), showed hippocampal volume deficits regardless of social context. However, the amygdala deficits were dependent upon both CORT and the mother being present, regardless of whether she exhibited maternal behavior or not (i.e., anesthetized). It is important to note that this study provides evidence that the amygdala’s dysfunction requires the co-occurrence of both increased CORT and a social context, while the hippocampus dysfunction relies on CORT in both social and non-social contexts. Additional experiments showed that temporarily eliminating the dysfunctional amygdala with microinjections of muscimol repaired social behavior to highlight a causal role for the amygdala in infant social behavior deficits (Rincón Cortés and Sullivan, 2010; Raineki et al., 2012, 2019; Opendak et al., 2020). The significance of these results is the identification of a combined social context-corticosterone pathology pathway that targets the amygdala, which was shown by replicating the necessary and sufficient conditions outside the nest that did not involve maternal behavior. Teasing apart the many deficits induced by maltreatment that are highly correlated with aberrant maternal behavior, is critical to identifying targeted interventions and treatments for early-life pathology.

## Conclusion

Research in children is the gold standard for understanding neurobehavioral development and a critical benchmark for understanding the neurobehavioral expression of pathology. However, while brain imaging techniques are powerful tools, resolution is low and models of inferential causation are frequently used instead of defining causal mechanisms within the brain, the organ of behavior. This combined with ethical limitations exploring the infant’s response to trauma limit our understanding of the developing brain. However, the emergence of robust animal research in brain networks to define neural circuits and extracellular and intracellular events, shows infants’ brains are indeed immediately altered in response to trauma and further altered by repeated trauma *via* brain programming. Non-human animal research fills a gap in our understanding of the child’s brain when clinically relevant questions are addressed and often builds bridges between basic and clinical domains that can transcend jargon and biases within research domains.

Within typical development, our current literature on bi-directional translational research in children and other infants of altricial species shows that frequent stress produces an increase in stress hormones, which is reduced by parental presence to inhibit responses to the threat. Animal models have shown the neural basis of this, highlighting a brain network involving suppression of the hypothalamic control of stress hormone release and inhibition of VTA dopamine (DA) release, to suppress amygdala plasticity molecules, and halting fear expression and learning (Opendak et al., 2019, 2021). Most importantly, in rodent adults CORT only modulates amygdala-dependent fear but serves as a switch to turn on and off fear in infants.

For atypical development, we reviewed the rodent literature with repeated infant adversity experiences illustrating this system of parental suppression of stress hormones, where the amygdala is greatly attenuated, and fear learning is not blocked. Furthermore, this dysfunctional system that is preserving high levels of stress in the infant when with the mother (failed social buffering), also disrupts social behavior with the mother.

Ultimately, this system initiates a pathological pathway of brain development that specifically targets and disrupts amygdala development to produce enduring dysfunctional processing of the mother within infant brain networks. We suggest that a better understanding of these age-specific roles of CORT and its dysfunction provides insight into age-specific identification of trauma effects related to elevated CORT, and potentially a better understanding of early markers of later-life pathology and mechanisms initiating the pathway to pathology.

The presence of the mother, even without exhibiting any form of behavior (i.e., anesthetized), reduced threat-induced stress increase in infants with adversity-rearing, and this is known as social buffering. The ability of the caregiver to socially buffer highlights the instrumental role of social context of adversity and how attachment to a caregiver can impact how an infant will react to trauma.

Deepening the understanding of typical and atypical development induced by repeated early-life adversity, especially within a social context, is crucial for designing age-specific interventional treatments that have efficacy in alleviating deleterious impacts of trauma during infancy on mental and physical health that are known to continue into adulthood. Early-life trauma, especially within the family, is emerging as one of the more potent factors of later-life psychopathology, and it is important to understand the mechanisms of action among risk factors that increase vulnerability to psychopathology. For reviews of this translational approach, please see Callaghan and Richardson (2011), Casey et al. (2011), Malter Cohen et al. (2013), Sarro et al. (2014), Callaghan B. et al. (2019), Meyer and Lee (2019), Opendak and Sullivan (2019), Patel et al. (2019).

## Author Contributions

NN, RMS, and RMZ: conceptualization, research, writing—original draft preparation, editing, and creating figures. SW, AU, BY, and AS: editing, research, and drawing figures. All authors have read and agreed to the published version of the manuscript.

## Conflict of Interest

The authors declare that the research was conducted in the absence of any commercial or financial relationships that could be construed as a potential conflict of interest.

## Publisher’s Note

All claims expressed in this article are solely those of the authors and do not necessarily represent those of their affiliated organizations, or those of the publisher, the editors and the reviewers. Any product that may be evaluated in this article, or claim that may be made by its manufacturer, is not guaranteed or endorsed by the publisher.
